# Mechanisms of Lin28-Mediated miRNA and mRNA Regulation—A Structural and Functional Perspective

**DOI:** 10.3390/ijms140816532

**Published:** 2013-08-09

**Authors:** Florian Mayr, Udo Heinemann

**Affiliations:** 1 Crystallography, Max-Delbrück Center for Molecular Medicine, Robert-Rössle Straße 10, Berlin 13125, Germany; E-Mail: florian.mayr@mdc-berlin.de; 2 Institute for Chemistry and Biochemistry, Freie Universität Berlin, Takustraße 6, Berlin 14195, Germany

**Keywords:** Lin28, *let-7* miRNA, miRNA processing, RNA-binding protein, cold-shock domain, zinc-knuckle domain, TUTase, oncogene, stem cell

## Abstract

Lin28 is an essential RNA-binding protein that is ubiquitously expressed in embryonic stem cells. Its physiological function has been linked to the regulation of differentiation, development, and oncogenesis as well as glucose metabolism. Lin28 mediates these pleiotropic functions by inhibiting *let-7* miRNA biogenesis and by modulating the translation of target mRNAs. Both activities strongly depend on Lin28’s RNA-binding domains (RBDs), an N-terminal cold-shock domain (CSD) and a C-terminal Zn-knuckle domain (ZKD). Recent biochemical and structural studies revealed the mechanisms of how Lin28 controls *let-7* biogenesis. Lin28 binds to the terminal loop of pri- and pre-*let-7* miRNA and represses their processing by Drosha and Dicer. Several biochemical and structural studies showed that the specificity of this interaction is mainly mediated by the ZKD with a conserved GGAGA or GGAGA-like motif. Further RNA crosslinking and immunoprecipitation coupled to high-throughput sequencing (CLIP-seq) studies confirmed this binding motif and uncovered a large number of new mRNA binding sites. Here we review exciting recent progress in our understanding of how Lin28 binds structurally diverse RNAs and fulfills its pleiotropic functions.

## 1. Introduction

Lin28 (cell lineage abnormal 28) is a conserved RNA-binding protein in higher eukaryotes that regulates several important cellular functions associated with development, glucose metabolism, differentiation and pluripotency. It was first described as a heterochronic gene in *Caenorhabditis elegans (C. elegans)*, since mutations within *lin-28* disturbed the developmental timing of the worm and accelerated differentiation of hypodermal seam cells and vulva stem cells [1,2]. Subsequent experiments revealed that Lin28 is expressed early in nematode embryonic and larval development, but its expression is down-regulated by *lin-4* and *let-7* miRNA as differentiation proceeds [2,3].

A similar expression pattern and physiological function was also shown for *Drosophila*, *Xenopus* and mammalian Lin28 [4]. The human paralogs Lin28a (routinely termed simply Lin28) and Lin28b encode for basic 23- or 28-kDa proteins that are highly expressed in embryonic stem cells (ESC) but are down-regulated upon differentiation of ESCs into embryoid bodies [5]. Reciprocally, Yu and colleagues used Lin28a, in conjunction with Oct4, Sox2 and Nanog, to reprogram adult human fibroblasts to induced pluripotent stem cells (iPSCs) [6]. A knockdown of Lin28a expression in mouse ESCs led to loss of Oct4 and Nanog expression, indicating an impaired self-renewal potential [7]. Increased Lin28a/Lin28b expression was reported in various hepatocellular and other carcinomas and was associated with poor patient prognosis [8–13]. Recently, Lin28a was linked to the regulation of developmental and metabolic processes. After ectopic overexpression of Lin28a mice developed a bigger size and delayed sexual maturation, whereas Lin28 knockout mice were smaller and died shortly after birth [14]. In addition, Lin28a overexpression was associated with increased insulin sensitivity and glucose metabolism, while a depletion of Lin28a resulted in insulin resistance and glucose intolerance [15].

**Figure 1:**
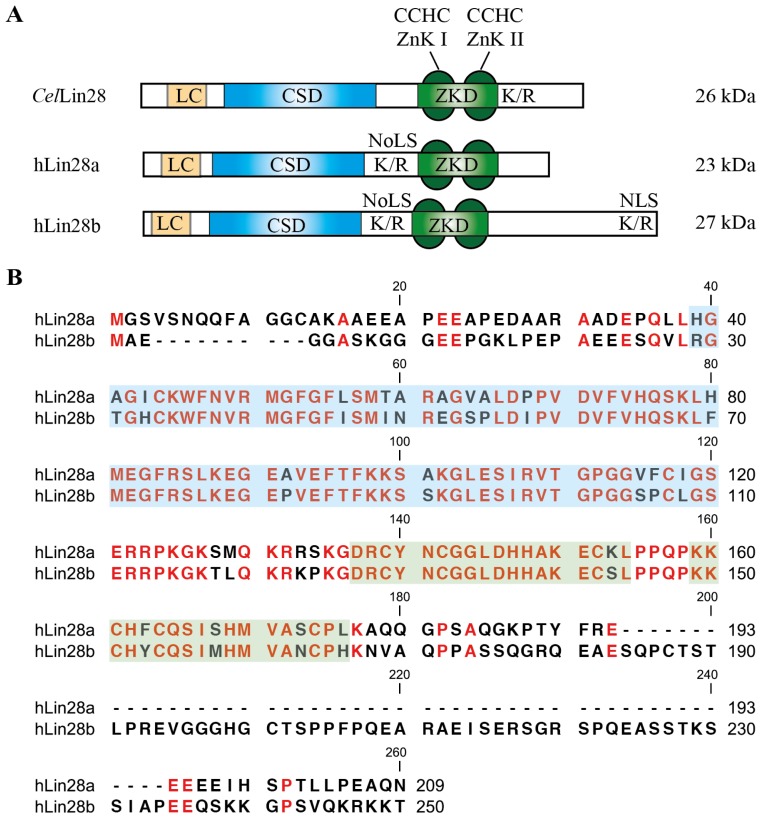
Domain organization of Lin28. (**A**) *Caenorhabditis elegans* (*Cel*) and human (h) Lin28a/Lin28b contain two RNA-binding domains (RBDs): an N-terminal cold-shock domain (CSD) and a C-terminal Zn-knuckle domain (ZKD) comprised of two retroviral type CCHC Zn knuckles (ZnK). Additionally, Lin28 harbors low-complexity sequences, Lys/Arg (K/R)-rich stretches, bipartite nuclear localization signals (NLS) or putative nucleolar localization sequences (NoLS); (**B**) Sequence alignment of hLin28a and hLin28B. Amino acids belonging to CSD or ZKD are shaded in blue or green, respectively.

On the molecular level, Lin28a and Lin28b act as both negative regulator of *let-7* miRNA biogenesis and post-transcriptional regulator of mRNA translation. Both activities strongly depend on Lin28’s two RNA-binding domains (RBDs): an N-terminal cold-shock domain (CSD) and a C-terminal Zn-knuckle domain (ZKD) composed of two tandemly arranged retroviral-type CCHC Zn knuckles. The individual domain combination of both RBDs is unique in animals with the RBDs being highly conserved. The human Lin28 paralogs share an overall sequence identity of 65% (FFAS, [16]) and contain low-complexity regions at the N-terminus, a putative bipartite nucleolar localization sequence (NoLS) as well as a C-terminal nuclear localization signal (NLS) in the case of Lin28b [17] (Figure 1). Lin28a and Lin28b can localize to both cytosol and nucleus [2,4,17–21] and interact with primary (pri-) or precursor (pre-) *let-7* miRNAs thereby preventing their maturation [20,22,23]. In addition, binding of Lin28a to messenger ribonucleoprotein complexes containing translation initiation (eIF3B, eI4E) and elongation factors (EF1α, EF1α2), poly(A) binding proteins, Igf2bps and RNA helicase A was reported in various studies [18,20,24,25]. Under stress conditions, Lin28a was shown to localize to cytoplasmic stress granules and P-bodies where mRNA translation is temporally stalled [18]. Since a mutation in Lin28a’s ZKD caused Lin28a to accumulate in the nucleus, it was suggested that Lin28a exits the nucleus in a complex with bound RNA and thus regulates the post-transcriptional processing of its target RNAs [18].

## 2. Lin28 Blocks *let-7* Processing

The opposing expression pattern of Lin28 and *let-7* miRNA became initially apparent when studying *C. elegans* larval development [2–4,26,27]. At an early stage in larval development both Lin28 and pri-*let-7* are present, however, no levels of either pre-*let-7* or mature *let-7* can be detected, indicating a regulation at a post-transcriptional level [28]. As larval development proceeds, a heterogenic cascade involving *lin-4* miRNA and the *let-7* sisters *mir-48/84/241* lead to a relief of pri-*let-7* processing inhibition and to a subsequent down-regulation of Lin28 expression (reviewed in [29]). This inverse relationship between Lin28 and *let-7* miRNA is also present in mammalian cells, where Lin28a/b are mainly expressed in undifferentiated cells, and mature *let-7* is only detectable upon differentiation or tissue development [5,20,23]. Furthermore, levels of pri-*let-7* remain constant throughout the entire differentiation or development process suggesting a negative regulation of *let-7* biogenesis by Lin28a/b in stem or progenitor cells [30–32]. Purification of pre-*let-7* bound complexes and subsequent analysis via mass spectrometry revealed that both human Lin28 paralogs specifically associate with pri- or pre-*let-7 in vivo* [20,22,23]. Moreover, *in vitro* purified Lin28a could inhibit pri- and pre-*let-7* processing by Drosha and Dicer by binding to the double-stranded stem close to the Dicer cleavage site and the pre-element (preE, terminal loop or hairpin) [33]. Mutations within Lin28’s CSD and ZKD impaired pre-*let-7* binding and inhibition of Dicer processing, suggesting a competitive relationship between Lin28 and Dicer [33–35]. Moreover, recent studies provided evidence that Lin28a/b can induce a structural change within pre-*let-7*’s preE, thereby leading to an opening of the double-stranded stem including the Dicer cleavage site [34–36].

Heo and colleagues revealed an additional inhibition mode of *let-7* miRNA processing, which irreversibly targets pre-*let-7* to a decay pathway [7,37]. They demonstrated that Lin28a/b induce oligo-uridylation of pre-*let-7*’s 3′ overhang. Oligo-uridylated pre-*let-7* is resistant to Dicer cleavage given that Dicer normally recognizes a 2-nt 3′ overhang in miRNAs via its PAZ domain. Thus, Dicer is unable to recognize the elongated 3′ overhang and to process pre-*let-7*. Furthermore, it was reported that oligo-uridylated RNAs recruit 3′–5′ exonucleases and are targeted for decay [38,39]. Indeed, oligo-uridylated pre-*let-7* was more rapidly degraded than unmodified pre-*let-7* [37]. Recently, Chang and colleagues identified the 3′–5′ exonuclease Dis3l2 that catalyzes the decay of oligo-uridylated pre-*let-7* in mouse ESCs [40]. Consistent with this, a knockdown of Dis3l2 in mouse ESCs caused an accumulation of uridylated pre-*let-7*. Oligo-uridylation of pre-*let-7* is catalyzed by the non-canonical poly(A) polymerase TUT4 (terminal uridyl transferase 4/Zcchc11) and to a minor extent by TUT7 (Zcchc6) in a Lin28-dependent manner [7,41,42]. Interestingly, these enzymes catalyze mono-uridylation of pre-miRNAs with a 1-nt 3′ overhang (like most pre-*let-7* family members) in the absence of Lin28, thereby enhancing Dicer-mediated processing [43]. However, in the presence of Lin28, pre-*let-7* and other miRNAs containing a GGAG motif within their preE were subjected to oligo-uridylation. Upon mutation of this motif, both Lin28 binding and oligo-uridylation were impaired, indicating that the GGAG motif is essential for these processes [7].

**Figure 2:**
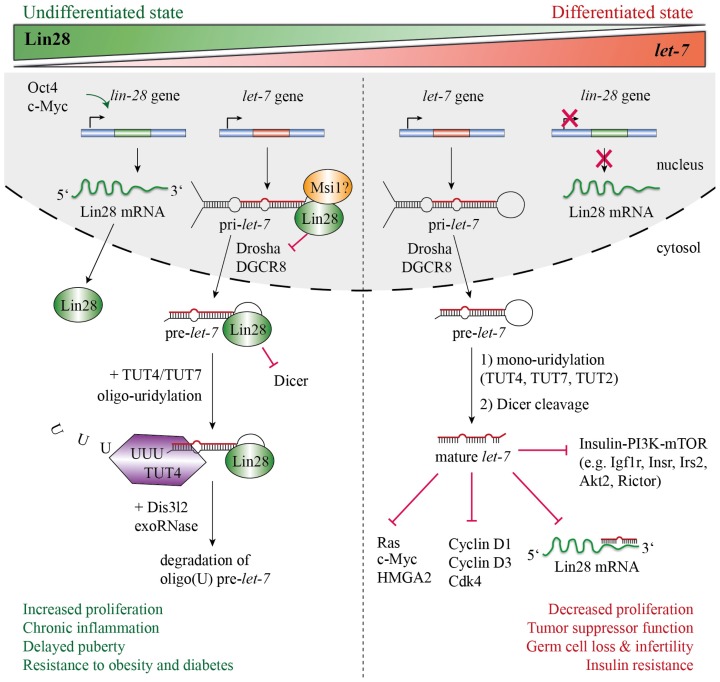
Lin28/*let-7* regulatory axis. In undifferentiated cells, Lin28 is highly expressed and blocks the biogenesis of *let-7* miRNA. By binding to the pre-element of pri- or pre-*let-7*, neither Drosha nor Dicer can process the corresponding *let-7* precursor. In addition, Lin28 recruits TUT4/TUT7 to pre-*let-7* and promotes its 3′-end oligo-uridylation. Oligo-uridylated pre-*let-7* cannot be cleaved by Dicer and thus serves as a signal for the cellular 3′–5′ exoribonuclease Dis3l2. Upon differentiation, Lin28 expression is reduced, which leads to increased levels of mature *let-7*. The latter silences gene expression of proto-oncogenes (Ras, c-Myc, Hmga2), cell cycle progression factors (Cyclin D1 and D3, Cdk4), components of the insulin-PI3K-mTOR pathway and Lin28 itself, thereby establishing a positive feedback loop. Besides its role in differentiation, a Lin28/*let-7* regulatory network is apparently involved in several cellular processes such as proliferation, oncogenesis, development and physiology, as well as metabolism (recently reviewed in [42]).

In *C. elegans* a similar mechanism for inhibiting pre-*let-7* processing has been reported [44]. The poly(U) polymerase PUP-2 was shown to oligo-uridylate pre-*let-7* in a Lin28-dependent fashion, thereby suppressing premature expression of mature *let-7* during larval development. In addition, subsequent RNA and chromatin immunoprecipitation assays revealed a specific interaction between Lin28 and pri-*let-7* that co-transcriptionally inhibits pri-*let-7* processing by Drosha [28]. An interaction between Lin28 and endogenous pri-*let-7* was also described for human ESCs and neuronal stem/progenitor cells [28]. Here, a highly expressed RNA-binding protein called Musashi1 (Msi1) selectively recruits Lin28a to the nucleus and synergistically blocks the cropping step of pri-*let-7* [45]. Moreover, it was suggested that Lin28b predominantly localizes to the nucleolus where it sequesters pri-*let-7*, thereby preventing Drosha processing in the nucleus [17]. Thus, Lin28a/b seem to obviate precocious expression of mature *let-7* during early development and differentiation by interfering with both the Drosha and Dicer complexes and by targeting pre-*let-7* towards degradation. Conversely, upon differentiation of stem or progenitor cells, *let-7* ensures constant down-regulation of Lin28 by binding to the 3′ UTR of Lin28 and its promoting transcription factor c-Myc [20] (Figure 2).

## 3. Lin28 Influences mRNA Translation

Besides regulation of *let-7* biogenesis, Lin28a/b can interact with various mRNAs and modulate their translation. Polesskaya and colleagues revealed that Lin28a can associate with polysomes and enhance translation of a number of mRNAs in differentiating myoblasts [25]. Among the first identified mRNA target was Igf2 (insulin-like growth factor 2), a major growth and differentiation factor in muscle tissue. Further evidence was provided that Lin28 recruits Igf2 mRNA to polysomes and enhances its translation via interactions with components of the translation initiation machinery. Subsequent studies revealed a number of additional mRNA targets of Lin28a in mouse ESCs such as H2a (histone 2a), Hmga1, Cyclin A, Cyclin B, Cdk4 and Oct4 [11,46–50]. An association of Lin28a with most of these mRNAs correlated with enhanced translation, suggesting that Lin28a maintains pluripotency by stimulating the translation of corresponding cell-cycle effectors. Further genome-wide studies revealed that Lin28a facilitates translation of genes important for growth and survival in human ESCs by recruiting RNA helicase A (RHA) to polysomes [24,51,52]. Additional mutagenesis studies revealed that the C-terminal part of Lin28a is required for RHA interactions, while mutations in the ZKD only impaired the stimulatory impact on translation, but not protein-protein interactions [46].

Very recently, a number of genome-wide Lin28 RNA crosslinking and immunoprecipitation coupled to high-throughput sequencing (HITS-Clip and PAR-CLIP) studies were conducted in human and mouse ESCs as well as somatic cells [19,49,53,54]. All of these studies have in common that only a small fraction of the identified RNA targets could be traced back to miRNAs, while the majority was mapped to thousands of mRNAs and ribosomal RNAs. For example, in mouse ESCs Lin28a was predominantly bound to mRNA transcripts (42%), mainly within the CDS and 3′ UTR. Furthermore, a gene ontology analysis of target RNAs, revealed a preferential interaction of Lin28a with mRNAs that are destined for the endoplasmic reticulum. Binding of Lin28a to these mRNAs was associated with a translational repression by reducing ribosome occupancy without affecting mRNA abundance [49].

**Figure 3:**
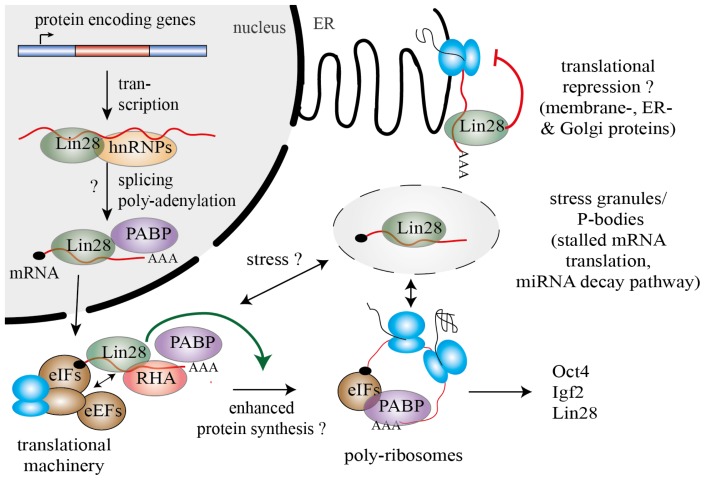
Lin28 binds various mRNAs and modulates their translation. Both Lin28 paralogs were shown to influence mRNA processing on several levels. In the nucleus, Lin28 could regulate splicing of bound pre-mRNAs in concert with heterogeneous nuclear ribonucleoproteins (hnRNPs). In the cytosol, Lin28 was shown to interact with an RNA helicase A (RHA) thereby modulating the translation of target mRNAs via interactions with eukaryotic translation initiation factors (eIFS), elongation factors (eEFS) and poly(A)-binding proteins (PABP). Furthermore, Lin28 was found to shuttle mRNAs to poly-ribosomes and, under stress condition, to P-bodies and stress granules, thereby providing a direct link to the miRNA decay machinery. Lin28 binding to mRNAs was typically associated with a globally enhanced protein synthesis. However, in hESCs Lin28 binding repressed translation of bound mRNAs that were destined for the ER.

On contrary, in human HEK293 cells, binding of Lin28a and Lin28b to its mRNA targets was linked to a globally enhanced protein synthesis [19,53]. As before in mESCs, both human Lin28 paralogs predominantly bound within exonic regions of mRNAs, thereby mirroring the predominant cytosolic localization of Lin28a/b in HEK293 cells. Among the top RNA targets were mRNAs encoding for splicing factors and RNA-binding proteins, cell-cycle regulators as well as Lin28 itself. Binding of Lin28b to its own mRNA, indeed, correlated with increased levels of Lin28b protein, thereby suggesting a *let-7* independent feed-forward mechanism to maintain high levels of Lin28b in proliferative cell types [19,53,54]. Apart from their own expression Lin28a/b also seem to drive expression of important cell-cycle regulators of the ERK signaling cascade, such as Cdk1, N-Ras, Ran and ERK. This would explain the strong proliferative defects observed upon Lin28b knockdown [53]. Wilbert and colleagues further detected widespread changes in protein levels of splicing factors upon down-regulation of Lin28a and Lin28b in human ESCs. Whereas Lin28a binding to hnRNP F mRNA repressed translation, binding to TDP-43 and FUS/TLS mRNA was associated with an enhanced protein synthesis of the corresponding transcript. Consistent with Lin28’s impact on alternative splicing factors, up-regulation of Lin28a in somatic HEK293 cells caused dramatic changes in alternative splicing patterns [54] (Figure 3).

## 4. Functional Importance of Lin28-Mediated mRNA and miRNA Regulation for Stem Cell Maintenance, Cancer and Development

The functional importance of Lin28 in stem cell maintenance and reconstituting pluripotency becomes apparent when looking at the signaling pathways in which Lin28a/b are involved. Both paralogs are highly expressed in mammalian ESCs and are a central part of a conserved pluripotency network. For example, the expression of Lin28a is driven by the proto-oncogenic transcription factors Oct4, Sox2 and Nanog with Sox2 being most critical for an efficient Lin28a expression [55,56]. Once Lin28a is expressed, it antagonizes *let-7* and hence de-represses *let-7* targets such as c-Myc, Sal4, Igf2bps, Hmga2, various cyclins as well as Lin28 itself, thereby ensuring a constant expression of stemness factors and cell cycle regulator [57]. In addition, Lin28a directly or indirectly stimulates the translation of mRNAs encoding for cell-cycle regulators or growth-promoting factors such as Cyclin A/B, Oct4 and Igf2 [18,25,48,50]. Consequently, Lin28a/b up-regulate the expression of cell-cycle regulators and growth-promoting factors via *let-7* dependent and *let-7* independent mechanisms, thereby activating and maintaining signaling pathways that are important for self-renewal and proliferation. In agreement with this, Lin28a overexpression is not essential for reprogramming human fibroblast to iPSCs but strongly accelerates reprogramming by stimulating cell proliferation [58].

The strong effect of Lin28a/b on cell progression and proliferation [59] and the frequent re-activation of Lin28a/b in multiple cancers [12] supported the role of Lin28 as a potential oncogene. Indeed, Lin28a/b overexpressing in NIH/3T3 cells led to tumor formation in nude mice and was linked to depletion of mature *let-7.* As a consequence, oncogenic *let-7* targets such as c-Myc and N-Ras were de-repressed, and, since c-Myc itself transcriptionally activates various oncogenic miRNAs as well as Lin28b, a positive feed-forward loop is established [12,60]. Iliopoulus and colleagues revealed another positive feedback loop between NF-κB, Lin28b, *let-7* and IL-6 (Interleukin 6). Transient activation of Src tyrosine kinase in immortalized breast cells led to activation of NF-κB, which binds to the Lin28b promoter and induces its expression. As a result, Lin28b represses *let-7* processing, the *let-7* target IL-6 can be produced and activate NF-κB, thereby closing the positive feedback loop [61]. Similar to its role in reprogramming somatic cells to iPSCs, an elevated expression of Lin28a/b might also be important in the formation of cancer stem cells (CSCs) [62]. This subpopulation of tumor cells is thought to be essential for the propagation of some cancer cells and might arise in a reprogramming-like mechanism [63]. Hence, Lin28a/b reactivation would contribute to the formation of metastasis thereby explaining why Lin28a/b up-regulation correlates with tumor aggressiveness and an advanced tumor stage [12,62].

Given that *let-7* family members target numerous metabolic genes, it is not surprising that Lin28a/b overexpression also has an impact on growth, developmental timing and metabolism. Using genome-wide association studies, genetic variations within the LIN28B loci was linked to changes of human height, timing of puberty and the age of menopause [64–66]. Consistent with these studies, Lin28a overexpression in transgenic mice led to similar phenotypes and was associated with increased insulin sensitivity and increased glucose uptake [14]. On the molecular level, Lin28a/b act on multiple components of the insulin-P13K-mTOR pathway, thereby explaining why administration of the mTOR inhibitor rapamycin could rescue the Lin28a-mediated metabolic phenotype [15]. Further *in vitro* studies showed that Lin28a de-represses *let-7* targets of the insulin-P13K-mTOR pathway such as Igf1r, Insr, Irs2, Akt2, Tsc1 and Rictor [15,67]. The authors could not rule out that Lin28a/b associate with these mRNAs itself and enhance their translation. Recent genome-wide Clip-seq studies indeed suggested that Lin28a/b binds to mRNAs of insulin and Igf receptors, glycolytic and mitochondrial enzymes thereby modulating their translation directly [19,52,53]. Hence, Lin28a/b seem to regulate both mRNA translation and *let-7* maturation to coordinate proliferative signaling pathways and cellular metabolism in order to maintain the self-renewal potential of stem or progenitor cells. However, given the wealth of recently identified mRNA targets of Lin28a/b, their overlap with known *let-7* targets and the interwoven signaling pathways, it remains to be determined which of the identified targets indeed contribute to the observed physiological functions.

## 5. Structural Basis for the RNA-Binding Specificity of Lin28

### 5.1. The Lin28 Zinc-Knuckle Domain Specifically Recognizes GGAG or GGAG-Like Motifs

After identifying *let-7* precursors as major targets of Lin28a and Lin28b, several groups aimed to identify the specificity of this interaction. Using electrophoretic mobility shift assays with different pre-*let-7* sequences, it became initially apparent that the terminal loop of pre-*let-7* (also called pre-element or preE) is sufficient for Lin28a binding [33]. An alignment of stem-loop precursors of *let-7* revealed a highly conserved GGAG motif within vertebrates that is critical for Lin28 binding. Mutations within this motif (GGAG→AAAG and GGAG→GUAU) released the Lin28a-mediated block of pri- or pre-*let-7* processing and impaired TUT4-mediated oligo-uridylation of pre-*let-7* [7,22]. On contrary, introduction of the GGAG motif into preE of an unrelated miRNA (pre-*miR-16-1*) allowed Lin28a binding and TUT4-mediated uridylation of this chimeric pre-miRNA [7].

**Figure 4:**
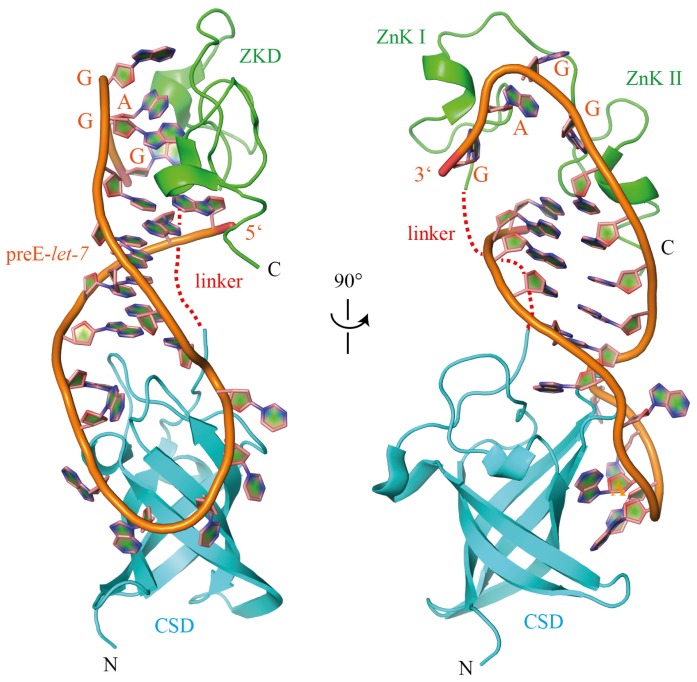
Co-crystal structure of a minimal mouse Lin28a construct with preE-*let-7*d derived RNA (PDB ID 3TRZ). The ZKD specifically binds to the conserved GGAG motif, whereas Lin28 CSD establishes extensive interactions with the less conserved terminal hairpin loop.

**Figure 5:**
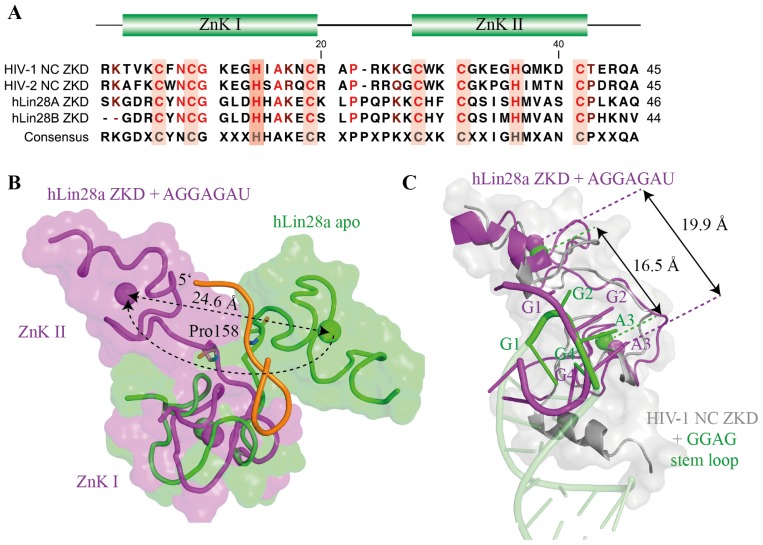

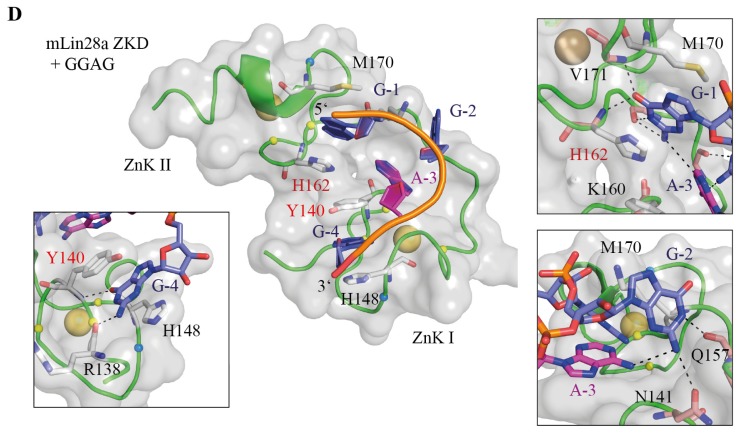
Lin28 ZKD specifically recognizes single-stranded GGAG or GGAG-like sequences. (**A**) Sequence alignment of HIV-1 NC, HIV-2 NC, hLin28a and hLin28b ZKDs. The chelating Cys and His residues of the CCHC Zn knuckles (ZnK) are shaded in red. Conserved residues are labeled from light red (100% type-conserved) to dark red (70% type-conserved); (**B**) Comparison between unbound hLin28a ZKD (green, PDB-ID 2CQF) and AGGAGAU-bound hLin28a ZKD (purple, PDB-ID 2LI8). Upon RNA binding, hLin28a ZKD undergoes a dramatic conformational shift mainly caused by a rotation of the Pro158 ψ angle; (**C**) In comparison to HIV-1 NC, the inter-knuckle linker of hLin28a ZKD harbors an additional Pro. As a consequence, the knuckles are further apart, thereby explaining why HIV-1 NC ZKD specifically binds G-2 and G-4 while hLin28a ZKD binds G-1 and G-4 of the GGAG motif in a hydrophobic pocket; (**D**) Structure of mLin28a ZKD bound to GGAG (derived from PDB-ID 3TSO). mLin28a is represented in green cartoon and the bound GGAG motif in purple (G) and pink (A). Tyr140 of the first and His162 of the second ZnK are key residues for the interaction, since they contact each other and stack with the bases, thereby establishing a kinked conformation in the RNA. All three guanosines are specifically recognized via various hydrogen bonds with backbone amide and carbonyl groups. In addition, G-1 and G-4 are bound in a hydrophobic pocket formed by His140, His162, Tyr140 and Met170.

Due to the close homology between Lin28’s ZKD and the ZKD of HIV-1 nucleocapsid protein (HIV NC), which was known to bind GGAG- or GGUG-containing loops within the HIV Ψ-RNA recognition element [68–70], it was suggested that Lin28’s ZKD mediates a specific interaction with the conserved GGAG motif (see Figure 5A). Indeed, mutations with Lin28’s ZKD specifically impaired pre-*let-7* binding as well as binding of the isolated Lin28 ZKD to GGAG-containing RNAs [7,34,35,71,72]. Co-crystal structures of a minimal mouse Lin28a construct with GGAG-containing oligonucleotides derived from preE-*let-7* (Figures 4 and 5) and a NMR solution structure of human Lin28a’s ZKD bound to AGGAGAU provided the final proof for the supposed interaction (Figure 5B,C) [35,72].

For Lin28:mRNA binding, so far no structural data has been obtained. However, despite their discrepancies in individual mRNA targets, most of the above mentioned genome-wide HITS-CLIP and PAR-CLIP studies identified GGAG or GGAG-like consensus motifs within Lin28a/b binding sites. For example, Wilbert *et al.* found a highly enriched GGAGA(U) consensus sequence that was enriched within loop structures [54]. Cho *et al.* detected AAGNNG, AAGNG and UGUG motifs that are often located in terminal loops of small RNA hairpins [49]. Finally, Graf *et al.* detected GGSWG (S = G or C, W = A or T) or AAGRWG (R = A or G) motifs in Lin28b binding sites. Using individual domain PAR-CLIP (iDo-PAR-CLIP) Graf and colleagues further confirmed the GGGAG sequence as the top motif within Lin28 ZKD binding sites, whereas Lin28 CSD binding sites were rather U-rich [53]. These data indicate that the GGAG motif is indeed the major determinant of Lin28 RNA binding and is recognized by the ZKD. Even a mutation of the first or second guanosine only moderately impairs the interaction, thereby mirroring the overall flexibility of both ZKD and RNA. A recent study revealed that CCHC Zn knuckles can be used to design single-stranded nucleic-acid binding proteins that specifically recognize a number of guanosines [73]. This study further demonstrated that the length of the inter-knuckle linker affects spacing between specifically bound guanosines. Hence, Lin28 ZKD probably prefers GNNG motifs over NGNG motifs as seen for HIV-1 NC ZKD (see Figure 5C). Interestingly, TUT4 and TUT7 also contain CCHC Zn knuckles that are critical for pre-*let-7* oligo-uridylation [42,74]. Compared to Lin28, the distance between these knuckles is larger (37 aa), indicating that they act independently from each other.

The second CCHC Zn knuckle undergoes a larger structural change upon RNA binding, whereby the central Zn^2+^ ion moves about 25 Å. Responsible for this large conformational shift is Pro158 within the Pro-rich linker region, since its ψ torsion angle performs a 130° rotation (Figure 5B) [72]. While HIV-1 NC ZKD specifically binds G-2 and G-4 of a GGAG tetraloop in a sequence-specific manner, each CCHC Zn knuckle of Lin28 specifically recognizes the first and fourth guanosine of the GGAG motif by sequence-specific hydrogen bonds to the bases. Hydrogen bonding is mediated by backbone carbonyl and amide groups of residues that are located within the rigid parts of the CCHC Zn knuckle. In addition to this sequence-specific interaction, both G-1 and G-4 are sandwiched in a hydrophobic pocket by one conserved Tyr and His in the first Zn knuckle and another conserved His and Met in the second Zn knuckle (Figure 5C). In the case of mLin28a:GGAG structures, G-2 is also bound in a sequence-specific manner via hydrogen bonds from backbone carbonyl groups and the N1 amino group of A-3. Even more, A-3 contributes to the formation of a strong kink within the RNA backbone, since it also contacts G-1 [35] (Figure 5D). Although such a strong bending of the RNA backbone was not observed in the hLin28a:AGGAGAU structure, the imposed structural changes within RNA and protein likely lead to a constant opening of neighboring double-stranded pre-*let-7* stem thereby masking the Dicer cleavage site [34,35].

### 5.2. The Lin28 CSD Has Broad Sequence Specificity and Can Induce Local Structural Changes within RNAs

Despite Lin28’s specificity for GGAG-containing RNAs, the isolated Lin28 ZKD is not sufficient for binding *let-7* precursors and blocking their processing [34,35]. So what is the contribution of Lin28’s CSD with respect to sequence specificity, binding affinity and inhibition of pre-*let-7* processing?

CSDs are highly conserved RBDs that are widely distributed in bacteria, animals and plants and fulfill pleiotropic functions mainly related to RNA metabolism (reviewed in [75]). Bacterial major cold-shock proteins (Csps) share between 30% and 45% sequence identity to Lin28 CSDs and are known to bind pyrimidine-rich ssDNA/ssRNA oligonucleotides with affinities in the sub-nanomolar to micromolar range [76–81]. In addition to this, they can act as RNA chaperones that destabilize local RNA secondary structures [82–84]. Crystal and NMR structures of Csps have been known since the 1990s [85–89].

A systematic binding analysis with *Xenopus tropicalis* (*Xtr*) Lin28b CSD revealed that this domain has a broad sequence specificity and shows the highest binding affinities for pyrimidine-rich RNA octamers that contain at least one guanosine at the 5′ end [34]. The observation was further confirmed by genome-wide PAR-CLIP studies, in which Lin28a/b binding sites were generally uridine-rich and flanked by one or more guanosines [19,53]. Moreover, these binding sites were typically located upstream of the corresponding ZKD binding sites, indicating a defined domain orientation of Lin28s’ RBDs on RNA targets [53].

**Figure 6:**
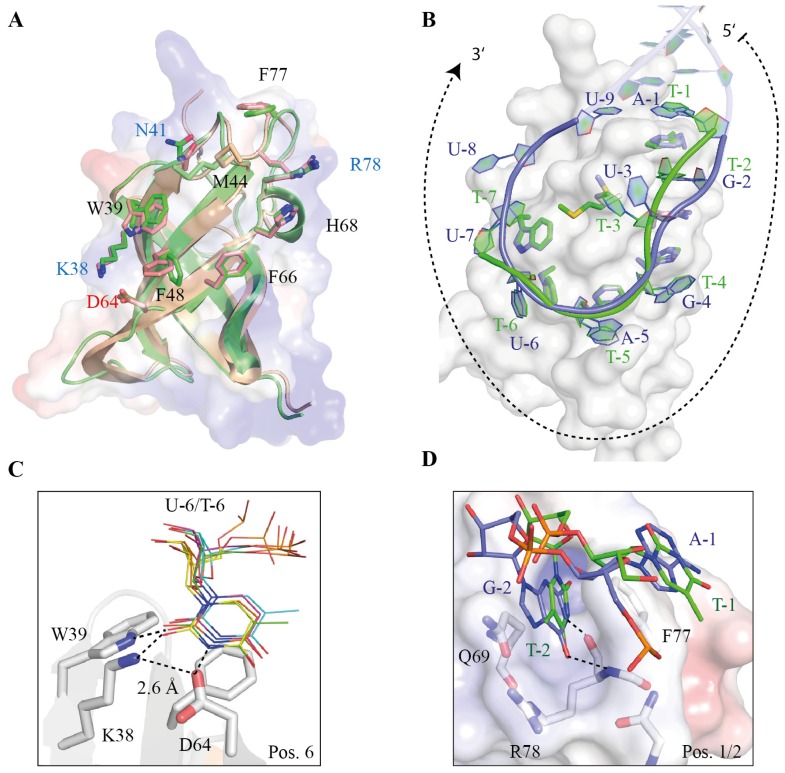
The Lin28 CSD can bind to a wide range of different RNA sequences. (**A**) Superimposition of unbound (skin color, PDB-ID 3ULJ) and heptathymidine-bound *Xtr*Lin28b CSD (green, PDB-ID 4A76). Both structures are highly conserved and reveal a pre-formed nucleic-acid binding platform with exposed aromatic residues; (**B**) Superimposition of *Xtr*Lin28b:dT_7_ (green) and mLin28s:preE-*let-7f* (RNA: blue, protein: gray, PDB-ID 3TS0). Both Lin28 CSDs bind single-stranded nucleic acids predominantly via base stacking interactions in a defined orientation. The protein nucleic-acid interaction surface is similar for binding subsites 1 to 7. Binding of an additional eighth (U-8) and ninth (U-9) base in mLin28:preE-*let-7f* is triggered by the formation of a closed RNA loop; (**C**) Superimposition of bound nucleotides at binding subsite 6 derived from various bacterial and Lin28 CSDs in complex with ssDNA/ssRNA (PDB-IDs 4A76, 4A75, 3TS0, 3TS2, 3PF4, 2HAX). All structures contained T or U nucleotides at this binding pocket. A highly conserved Lys-Asp salt bridge limits the size of the pocket and establishes specific hydrogen bonds with the T/U base; (**D**) Since few interactions are formed with the sugar-phosphate backbone, the bound oligonucleotides can adopt different backbone conformations to optimize binding with Lin28 CSD. For example, at binding subsite 2, the sugar-phosphate backbone of mLin28a:preE-*let-7f* is farther displaced from the protein, thereby enabling binding of G (G-2) instead of T (T-2) without disrupting hydrogen bonds.

Co-crystal structures of Lin28 CSDs in complex with ssDNA and preE-*let-7* derived RNA stem loops provided valuable information about Lin28’s specificity and function in pre-*let-7* and mRNA binding [34,35]. Lin28 CSDs bind to single-stranded nucleic acids via a conserved nucleic acid-binding platform mainly formed of exposed aromatic residues. Unlike for Lin28 ZKD, this binding platform is already pre-formed in the apo protein and, consequently, only subtle changes are observed upon nucleic-acid binding (Figure 6A). Binding of ssDNA and ssRNA are remarkably similar and dominated by π-stacking interactions with exposed aromatic residues (Figure 6B). Consistent with solution binding experiments and bacterial Csp:ssDNA/RNA structures [79,90], Lin28 CSD binds up to 8 nucleotides arranged in a curved single strand with defined orientation. In the case of the mLin28a:preE-*let-7* structures, an additional ninth nucleotide is visible that establishes hydrogen bonds with the first base, thereby closing the preE stem loop. Sequence-specific binding is mainly mediated at position 6, since the presence of a conserved Lys-Asp salt bridge limits the flexibility and, consequently, the size of the binding pocket and contributes to specific hydrogen bonds with the U/T base. In addition to this, at binding subsite 2 either a T, U or G is specifically recognized within a hydrophobic pocket. Despite the difference in size, the corresponding bases are recognized by similar hydrogen bonds. The lack of contacts with the CSD allows the DNA/RNA backbone to adopt slightly different conformations without disturbing hydrogen bonding.

**Figure 7:**
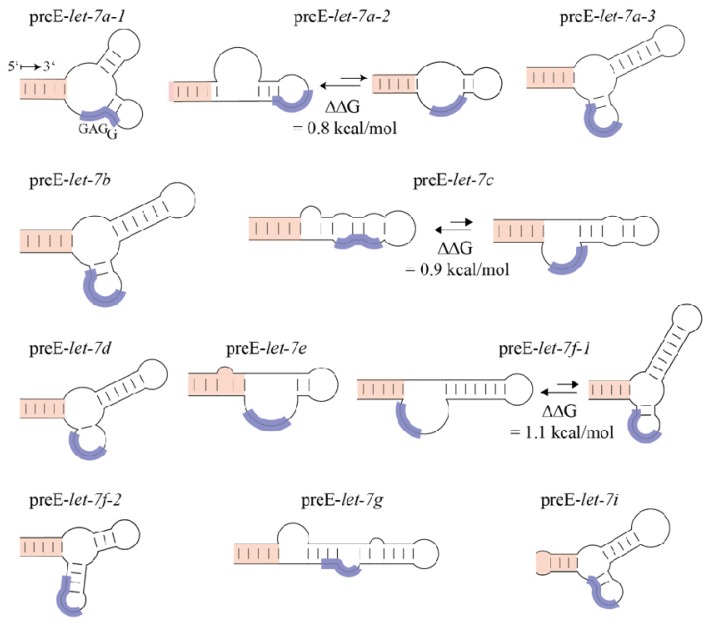
The pre-elements of *let-7* family members are structurally diverse. In six out of eleven human *let-7* family members, the conserved GGAG motif (blue) is inaccessible for ZKD binding in the lowest-energy folding state. Secondary structure predictions of human *let-7* family members (except miR-98 and miR-202) were calculated and visualized by CLC genomics workbench 3.65. All lowest-energy structures within a ΔΔG range of 1.5 kcal/mol are depicted. For simplicity, only 5 bp of the miRNA stem are shown (labeled in red).

Besides its contribution to binding affinity and specificity, Lin28 CSD can affect and reorganize secondary structures within RNA targets. The first hint in this direction came from a study that examined the effect of Lin28a binding on pre-*let-7g* secondary structure using enzymatic foot-printing [36]. Upon Lin28a binding, some regions of preE as well as a part of the double-stranded stem of pre-*let-7* became more susceptible to cleavage by single-strand-specific ribonucleases. Hence, the authors concluded that Lin28a is able to unwind the double-stranded stem of pre-*let-7*, thereby blocking the Dicer cleavage site. Second, Nam *et al.* provided evidence that Lin28a’s CSD can partially melt double-stranded stem loops to generate an optimal binding interface [35]. Third, using site-directed mutagenesis in combination with a kinetic analysis of *Xtr*Lin28b-mediated remodeling of pre-*let-7g*, it was shown that Lin28’s CSD first binds to pre-*let-7* and induces a structural change [34]. Consistent with earlier studies on bacterial Csps [83,91], highly conserved His (His68 in *Xtr*Lin28b-binding subsite 4) and Phe residues (Phe77-binding subsite 1,2) were crucial for the remodeling reaction. The CSD-induced remodeling might be important for proper recognition of the GGAG motif by Lin28’s ZKD, since in most pre-*let-7* structures the conserved GGAG motif is involved in secondary structures and therefore not accessible for binding (Figure 7). Genome-wide PAR-CLIP and HITS-CLIP studies further supported this hypothesis, since Lin28a/b could recognize RNA binding sites that are predicted to be involved in stable secondary structures [19,49]. Such a chaperone-like function of Lin28 might be an important regulatory mechanism that allows downstream RBPs either to dissociate from or associate with RNPs and influence their processing. Most notably, a recent study provided evidence that Dis3l2 exoribonuclease degrades oligo-uridylated pre-*let-7*. This enzyme is composed of one ribonuclease II domain, two CSDs and one CSD-like S1 domain. Interestingly, both the CSDs and the S1 domain were essential for Dis3l2 binding and degradation of oligo-uridylated pre-*let-7*. Given the preference of Lin28 CSD’s for U-rich binding sites, this suggests that the CSD might recognize the oligo(U) tail. In addition, it may assist in the exoribonucleolytic degradation of oligo(U)-pre-*let-7* by partially unwinding the double-stranded miRNA stem.

## 6. Summary and Conclusions

Recent structural and biochemical studies along with genome-wide CLIP-seq studies have greatly improved our understanding of how Lin28 recognizes target RNAs and fulfills its pleiotropic functions related to regulation of miRNA and mRNA processing as well as mRNA translation. In the case of *let-7* biogenesis, Lin28 ZKD specifically recognizes a conserved GGAG motif within preE-*let-7* and induces a strong bending of the bound RNA backbone. Thus, the adjacent Dicer cleavage site remains constantly unwound and pre-*let-7* cannot be processed anymore. Apart from a minor preference for pyrimidine-rich sequences with one flanking guanosine, the CSD did not reveal any clear sequence specificity, but was able to remodel local RNA secondary structures. This might be important in three ways. First, initial binding of the CSD to single-stranded RNA sequences can induce a conformation in which the GGAG motif is accessible for subsequent ZKD binding. Second, the CSD might trigger structural changes within target RNAs thereby stimulating downstream processes such as pre-*let-7* oligo-uridylation. Third, the wide RNA-binding specificity of the CSD enables Lin28 to recognize all *let-7* family precursors in a defined 5′–3′ orientation despite the low sequence conservation within *let-7* preEs. Consequently, Lin28 impairs *let-7* biogenesis and irreversibly targets pre-*let-7* to degradation.

On the mRNA level the combination of both domains enables Lin28a/b to bind thousands of mRNAs. Although there is still no consensus how Lin28 mRNA binding influences mRNA processing and regulates translation, the observed principles with respect to sequence specificity and RNA remodeling are also valid here. Lin28a/b recognize GGAG or GGAG-like motifs and can access these motifs even if they are embedded in predicted secondary structures. The CSD typically binds to uridine-rich regions upstream of the ZKD binding site and is responsible for Lin28’s RNA chaperone-like function. The defined orientation of Lin28 on bound RNA might allow a specific recruitment of downstream factors, such as RNA helicase A. However, downstream effects of Lin28:mRNA binding were quite distinct in recent genome-wide CLIP-seq studies and comprised translational stimulation of growth-promoting and alternative splicing factors, as wells as translational repression of ER-destined mRNAs [19,49,52–54]. In addition to the localization of binding sites (coding sequence, 3′UTR), Lin28-induced structural changes within mRNAs and/or direct protein-protein interactions might affect the translation efficiency of target mRNAs.

Therefore, it will be essential to understand how Lin28a/b regulate mRNA processing and translation in more detail. Ongoing studies will need to determine which cellular factors are involved in these processes and which factors regulate the activity of Lin28a/b within different cellular compartments and cells/tissues. Furthermore, it remains to be verified which of the huge number of novel mRNA targets are indeed directly regulated by Lin28a/b and what are their impact on stem cell maintenance/differentiation, development, metabolism and cancer. Last but not least, additional structural and functional data of how Lin28 binds mRNA targets and interacts with downstream components such as RNA helicase A may help to elucidate the mechanisms behind Lin28-mediated translational enhancement. Although much effort has been undertaken to unravel the molecular mechanisms that control the Lin28/*let-7* regulatory axis, there are still a number of issues that remain to be solved. Which precise mechanisms does Lin28 use to inhibit pri-*let-7* processing by the Microprocessor in the nucleus? How does Lin28 stimulate TUT4/TUT7-mediated oligo-uridylation of pre-*let-7*? And is the observed RNA-chaperone-like function of Lin28 mandatory for fulfilling its tasks? Understanding these issues might help us to exploit Lin28’s function and manipulate the involved pathways for improved tissue re-engineering and novel treatments of cancer or metabolic diseases.
